# Effect of monosodium l-glutamate (umami substance) on cognitive function in people with dementia

**DOI:** 10.1038/s41430-018-0349-x

**Published:** 2018-10-22

**Authors:** Minoru Kouzuki, Miyako Taniguchi, Tetsuya Suzuki, Masaya Nagano, Syouta Nakamura, Yuto Katsumata, Hideki Matsumoto, Katsuya Urakami

**Affiliations:** 1 Department of Biological Regulation, School of Health Science, Faculty of Medicine, Tottori University, 86 Nishicho, Yonago, 683-8503 Japan; 2 Work Life Balance Support Center, Tottori University Hospital, 36-1 Nishicho, Yonago, 683-8504 Japan; 3 Frontier Research Laboratories, Institute for Innovation, Ajinomoto Co., Inc., 1-1, Suzuki-Cho, Kawasaki-Ku, Kawasaki-Shi, 210-8681 Japan

**Keywords:** Nutritional supplements, Dementia

## Abstract

**Background/objectives:**

This study assessed the effect of continuous ingestion of monosodium l-glutamate (MSG) on cognitive function and dietary score in dementia patients.

**Subjects/methods:**

This was a single-blind, placebo-controlled trial involving 159 subjects with dementia residing in a hospital or nursing home. We assigned the subjects to a group that ingested MSG thrice daily (0.9 g/dose) (MSG group; *n* = 79) or a group that ingested NaCl thrice daily (0.26 g/dose) (Control group; *n* = 80). This study consisted of a 12-week intake period, followed by a 4-week follow-up period without the ingestion of MSG or NaCl. We performed physical examination, cognitive symptom tests (the Touch Panel-type Dementia Assessment Scale (TDAS) and Gottfries–Bråne–Steen Scale (GBSS)), palatability and behaviour questionnaires, and blood tests before and after the intervention and after the follow-up period.

**Results:**

There were no significant differences in the TDAS and GBSS total scores between the groups before and after the intervention. However, regarding the TDAS sub-items, “the accuracy of the order of a process” did not deteriorate in the MSG group compared with that observed in the Control group (*p* < 0.05). At the follow-up assessment, the TDAS total scores in the MSG group showed significant improvement compared with those reported in the Control group (*p* < 0.05). Furthermore, there was a correlation of changes from pre-intervention to post-intervention between the TDAS and enjoyment of the meal (*r* = −0.299, *p* = 0.049).

**Conclusions:**

Our results suggest that continued ingestion of MSG has an effect on cognitive function. Furthermore, the patients with improved questionnaires about palatability survey showed greater improvement in cognitive function.

## Introduction

Dementia is a cognitive disorder primarily affecting the elderly [1, 2]. In recent years, the incidence of dementia has increased rapidly with populations aging worldwide, and according to a 2013 report, the number of patients with dementia worldwide is expected to reach 65.7 million in 2030 and 115.4 million in 2050 [3].

Recently, attempts to prevent dementia have led to an interest in dietary therapy. One study observed that frequent consumption of fruits and vegetables might decrease the risk of dementia [4] as these foods have protective effects that may be attributed to antioxidant compounds. Another study reported that elderly individuals who consumed fish or seafood, which are particularly rich in anti-inflammatory and antioxidant omega-3 polyunsaturated fatty acids, once per week had a lower risk of developing dementia [5]. Other studies reported reduced risks of developing dementia with consumption of light-to-moderate alcohol [6], green tea [7] and Mediterranean diet [8]. Further studies found that exercise [9], intellectual activities [10], and aromatherapy [11], which activate the brain, were useful in decreasing dementia and improving cognitive function; in other words, effects of adjusting the environment in the brain or the continuous transmission of sensory signals to the brain help to prevent cognitive decline. However, evidence on the effects of non-pharmacological interventions on the cognitive symptoms of patients with moderate or severe dementia is currently limited.

Monosodium l-glutamate (MSG) has been used for over a century as a seasoning that provides umami, which has been established as the fifth basic taste (following sweet, salty, sour, and bitter) with potential effects on taste and appetite of human. Decreased sensitivity to umami and increased morbidity related to taste disorders are known to occur with ageing [12]. This may be attributed to the lower levels of zinc observed in the serum of elderly individuals compared with those observed in younger individuals [13, 14]. MSG increases the secretion of saliva from the parotid glands [15] and gastric juice [16] by transmitting taste signals to the brain via oral or gastric receptors, thus promoting digestion [17]. Therefore, intake of MSG may increase the absorption of zinc from the intestine and enhance the taste of meals. Furthermore, input from glutamate receptors on the tongue may be processed in the primary taste cortex [18]; reports have used functional MRI to demonstrate that the umami taste’s stimuli activate the taste cortical areas [19]. Therefore, it is hypothesised that ingestion of MSG may activate the gut–brain axis.

Continuous supplementation of MSG improved the quality of life and nutritional status of hospitalised elderly patients by increasing the levels of reduced-form albumin [20, 21]. However, the effect of continued MSG-related transmission of taste signals to the brain on the cognitive function of patients remains unclear. In addition, there is a lack of studies investigating the effects of prolonged intake of MSG in patients with dementia. This study assessed the effects of continuous ingestion of MSG on cognitive symptoms and dietary patterns in patients with dementia.

## Subjects and methods

### Subjects

Between September 2014 and November 2015, 173 subjects with dementia were recruited from hospitals or nursing homes (e.g. geriatric health service facilities, special nursing homes for the aged, and group homes). Although the severity of dementia was not examined in this study, it may be inferred that the subjects suffered from at least moderate dementia because the residents of these facilities have a low degree of independence in their everyday lives [22]. Fourteen subjects who had previously diagnosed taste-related diseases, and/or who had received chemotherapy within the past year, and/or who had undergone dietary therapy for hypertension, and/or who had not previously been diagnosed with dementia were excluded. A total of 159 subjects were included in the study. The design of this study was approved by the ethics committee of Tottori University (Yonago, Japan) and Ajinomoto Co., Inc., (Tokyo, Japan). The study was registered in the UMIN-Clinical Trials Registry (UMIN000020573). The research protocol was explained to the patients and guardians, who provided informed consent for participation.

### Procedures

This was a single-blind, placebo-controlled trial. Assessments included measurements of height and weight; the Touch Panel-type Dementia Assessment Scale (TDAS) (Nihon Kohden Corporation, Tokyo, Japan) [23]; Gottfries–Bråne–Steen Scale (GBSS) [24]; questionnaires regarding daily performance and dietary survey; and blood tests prior to the intervention. We performed stratified randomisation considering total TDAS score, total GBSS score, gender and age. Subjects were assigned to receive three daily ingestion of MSG (MSG group, *n* = 79) or NaCl (Control group, *n* = 80) for 12 weeks, consistent with the timeline of a previous study [21]. Following a 12-week dietary intervention, we repeated the above-listed examinations prior to a 4-week follow-up period in which the patients did not ingest MSG or NaCl. The examinations were repeated again after the follow-up period. Subjects who discontinued either the 12-week dietary intervention or 4-week follow-up were excluded from further analysis; these included subjects who failed to ingest MSG or NaCl, and/or died/left the facility after the pre-intervention testing.

### Diet

The amounts of ingested MSG and NaCl were based on those reported in a previous study [21]. Specifically, 0.9 g/dose MSG or 0.26 g/dose NaCl (equivalent to Na in the MSG molecule) were added to three meals per day (breakfast, lunch and dinner). When applicable, MSG or NaCl was added to rice porridge, miso soup, or other soup; otherwise, these additives were mixed in the main dish. The intake of meals containing MSG or NaCl was evaluated by the nursing caregiver. Subjects who ingested MSG or NaCl for less than three quarters of the intervention period, or who ingested these substances beyond the intervention period were excluded. In this study, daily meals were prepared by the staff of the facility under supervision by a nutritionist.

### General observation of subjects

We measured the height and weight of each patient to calculate the body mass index (BMI). Blood tests and observation of the defecation conditions were performed to evaluate the nutritional status of subjects. The levels of zinc and total protein in the serum were measured by LSI Medience Corporation (Tokyo, Japan). Pre-intervention and post-intervention and post-follow-up blood samples were collected at the same time of the morning; samples were not collected from difficult cases or patients who refused the examination. Defecation conditions, particularly the frequency of defecation and incidence of diarrhoea, were assessed daily by caregivers who primarily provided nursing care to the subjects.

### Cognitive symptom tests

To assess the cognitive symptoms, we used the TDAS as a subjective evaluation method and GBSS as an objective evaluation method. The TDAS is a modified version of the Alzheimer’s disease Assessment Scale (ADAS) [25] in which subjects enter their answers directly into a touch panel-type computer following instructions. The nine examination items include ‘word recognition’, ‘following a command’, ‘visual-spatial perception’, ‘accuracy of the order of a process’, ‘naming fingers’, ‘orientation’, ‘money calculation’, ‘object recognition’ and ‘clock time recognition’. Scores range from 0 (all correct answers) to 101 points (all incorrect answers). Notably, TDAS could not be used in difficult cases of severe cognitive dysfunction.

The GBSS, an objective evaluation method of cognitive symptoms, is comprised of four subscales: ‘motor function’, ‘intellectual function’, ‘emotional function’ and ‘symptoms common in dementia syndromes’. Each item is scored on a seven-point scale (0–6 points). If rating is not possible, the item is scored as 9 points. Higher scores indicate more severe cognitive dysfunction. The GBSS was completed by the primary nursing caregivers of the subjects.

### Palatability and behaviour questionnaire

We also administered a questionnaire regarding palatability and daily performance. Three items (enjoyment of the meal, deliciousness of the meal and strength of flavour) were used to enquire about palatability of the foods. Each item was scored on a five-point scale (from 0 to 4 points indicating the worst and best responses, respectively). There were also some subjects who did not answer. Daily performance was assessed using 10 items described in a previous study [21]. The assessment of daily performance was performed by the primary nursing caregivers of the subjects.

### Statistics

The sample size was originally defined as 200 patients (100 in each arm) on the basis of feasibility considerations. However, because of difficulties in the recruitment process, the sample size was reduced to 159. We were unable to infer the effect size in the calculation of the sample size due to the lack of previous research evaluating the effect of MSG on cognitive symptoms. Therefore, we did not perform a power analysis in this study.

The SPSS statistical software (version 23, IBM Japan, Tokyo, Japan) was used for statistical analyses. The Shapiro–Wilk test was used to assess the normal distribution of the data. The Levene’s test was used to assess the equality of variances. Differences in baseline demographics and background characteristics of subjects were assessed using a Student’s *t*-test, Welch’s *t*-test, Mann‒Whitney *U* test, or chi-squared test. Mean changes from baseline for each measured outcome were compared between groups using a Student’s *t*-test, Welch’s *t*-test or Mann‒Whitney *U* test. The Bonferroni correction was used for multiple comparisons of the degree of change in each group. The Wilcoxon signed-rank test was used to compare the frequency of defecation and diarrhoea between the first 4 weeks and last 4 weeks of the dietary intervention period. The Spearman’s correlation coefficient was used to evaluate the correlations between TDAS scores and the results of the dietary survey. All statistical significance tests were two-sided, and an alpha-level of 0.05 was considered statistically significant.

## Results

### Subjects and baseline characteristics

**Fig. 1:**
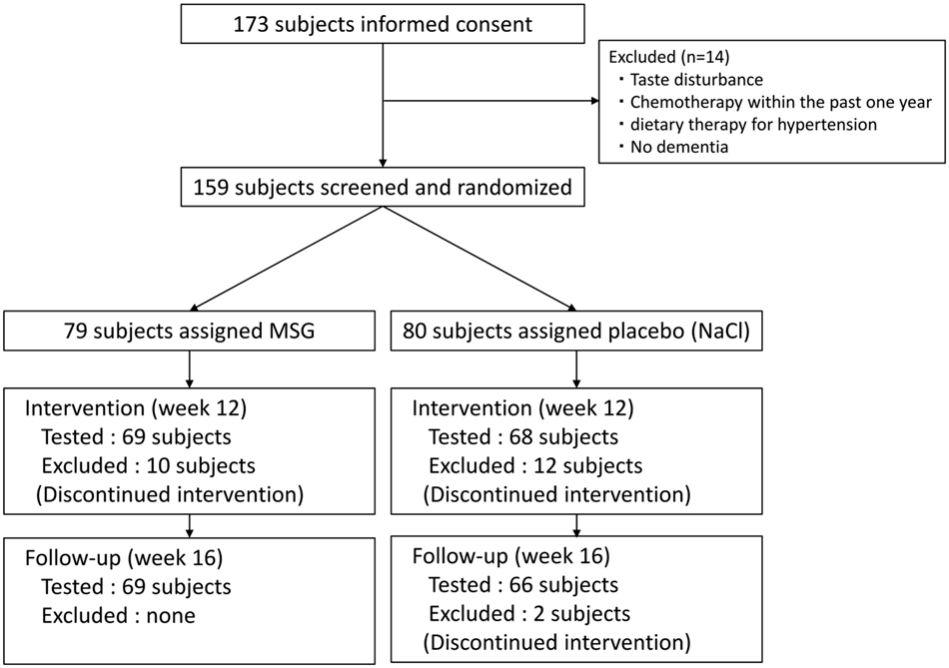
Flow chart of the subjects of this study. MSG monosodium l-glutamate

Figure 1 shows the subjects flow through in this study. A total of 159 subjects underwent pre-intervention assessment. Following the 12-week intervention, 137 subjects remained eligible for post-intervention assessment. Of these, 69 and 66 subjects in the MSG and Control groups, respectively, remained eligible for post-follow-up assessment. In the MSG group, 41 subjects had Alzheimer’s disease (AD), three had vascular dementia, two had dementia with Lewy bodies (DLB), and 23 had unspecified dementia. In the Control group, 46 subjects had AD, three had DLB, and 17 had unspecified dementia. The characteristics of the subjects are summarised in Table 1. The groups did not differ in terms of age, sex, TDAS scores, or GBSS scores.

**Table 1:** Baseline characteristics of subjects

Variable	MSG	Control	*p-*value
Number (*n*)	69	66	–
Age (yr)	86.5 ± 0.75	87.8 ± 0.65	0.185
Gender (M:F)	8:61	9:57	0.721
Type of dementia (*n*/%)			
Alzheimer’s disease	41/59	46/70	–
Vascular dementia	3/4	0/0	–
Dementia with Lewy bodies	2/3	3/4	–
Unspecified dementia	23/34	17/26	–
Heigh (cm)	147.3 ± 0.87	147.1 ± 1.13	0.764
Weight (kg)	46.9 ± 0.89	46.7 ± 1.12	0.883
BMI (kg/m^2^)	21.7 ± 0.44	21.5 ± 0.43	0.791
TDAS	56.5 ± 3.35	53.8 ± 3.87	0.610
GBSS	64.0 ± 3.94	65.5 ± 3.95	0.794

Data presented as mean ± standard error (SE)

In height, weight, and BMI, *n* = 62 (control group) and *n* = 66 (MSG group)

In TDAS, *n* = 42 (Control group) and *n* = 44 (MSG group)

*BMI* body mass index, *TDAS* Touch Panel-type Dementia Assessment Scale, *GBSS* Gottfries–Bråne–Steen Scale, *MSG* monosodium l-glutamate

### General observation of subjects

Table 2 lists the BMI values and blood test results of the subjects. The MSG group exhibited significant post-intervention increases in BMI (*p* < 0.05). Changes in BMI differed significantly between the groups after the intervention (*p* = 0.008). Furthermore, an analysis of intra-group fluctuation in blood tests revealed that after intervention, the levels of total protein increased in the Control group (*p* < 0.05), while the levels of zinc increased in the MSG group (*p* < 0.05).

**Table 2:** Results of the blood test, physical examination, palatability survey and daily performance

Test item	group	Baseline		Intervention		Follow-up	
		Mean ± SE	*p-* value^a^	Mean ± SE	*p-* value^b^	Mean ± SE	*p-* value^b^
*Blood test*							
Total protein (g/dL; reference 6.7–8.3)	Control	6.8 ± 0.1	0.564	7.0 ± 0.1^c^	0.273	6.9 ± 0.1	0.231
	MSG	6.9 ± 0.1		7.0 ± 0.1		6.9 ± 0.1	
Zinc (μg/dL; reference 64–111)	Control	63.5 ± 1.5	0.404	64.8 ± 1.4	0.177	65.3 ± 1.4	0.643
	MSG	61.8 ± 1.4		65.7 ± 1.3^c^		65.1 ± 1.7	
*Physical examination*							
BMI (kg/m^2^)	Control	21.5 ± 0.4	0.838	21.6 ± 0.4	0.008	21.5 ± 0.4	0.108
	MSG	21.7 ± 0.4		22.1 ± 0.4^c^		22.0 ± 0.4	
*Palatability survey*							
Enjoyment of the meal	Control	3.33 ± 0.09	0.909	3.27 ± 0.10	0.919	3.47 ± 0.10	0.687
	MSG	3.36 ± 0.09		3.32 ± 0.08		3.50 ± 0.08	
Deliciousness of the meal	Control	3.65 ± 0.08	0.842	3.67 ± 0.09	0.499	3.70 ± 0.08	0.906
	MSG	3.59 ± 0.09		3.61 ± 0.08		3.77 ± 0.06	
Strength of flavour	Control	3.25 ± 0.13	0.864	3.10 ± 0.13	0.022	3.32 ± 0.12	0.382
	MSG	3.20 ± 0.14		3.54 ± 0.09		3.58 ± 0.12	
*Daily performance*							
Response when called	Control	3.22 ± 0.13	0.983	3.10 ± 0.14	0.297	3.11 ± 0.15	0.688
	MSG	3.26 ± 0.12		3.25 ± 0.13		3.19 ± 0.14	
Voice (volume and distinction)	Control	3.15 ± 0.14	0.360	3.12 ± 0.12	0.460	3.23 ± 0.12	0.700
	MSG	3.33 ± 0.13		3.14 ± 0.14		3.45 ± 0.13	
Understanding of simple conversation	Control	2.69 ± 0.14	0.972	2.62 ± 0.15	0.605	2.64 ± 0.16	0.483
	MSG	2.70 ± 0.14		2.74 ± 0.14		2.59 ± 0.15	
Facial expression	Control	2.96 ± 0.14	0.584	2.76 ± 0.14	0.483	2.80 ± 0.14	0.562
	MSG	3.09 ± 0.13		3.04 ± 0.12		3.12 ± 0.12	
Concentration on conversation	Control	3.28 ± 0.16	0.041	3.28 ± 0.16	0.250	3.50 ± 0.14	0.065
	MSG	3.70 ± 0.10		3.59 ± 0.10		3.70 ± 0.10	
Concentration on eating during mealtime	Control	3.32 ± 0.11	0.371	3.37 ± 0.11	0.084	3.44 ± 0.11	0.191
	MSG	3.46 ± 0.10		3.42 ± 0.09		3.44 ± 0.11	
Motivation to eat	Control	3.31 ± 0.14	0.666	3.25 ± 0.14	0.693	3.30 ± 0.14	0.370
	MSG	3.33 ± 0.12		3.41 ± 0.11		3.51 ± 0.10	
Maintenance of posture during mealtime	Control	3.32 ± 0.12	0.766	3.31 ± 0.14	0.523	3.32 ± 0.13	0.089
	MSG	3.23 ± 0.15		3.48 ± 0.12		3.55 ± 0.11 ^d^	
Activities of eating	Control	3.12 ± 0.15	0.733	3.07 ± 0.16	0.998	3.17 ± 0.15	0.575
	MSG	3.03 ± 0.16		3.04 ± 0.17		3.10 ± 0.16	
Understanding of close relatives	Control	2.65 ± 0.15	0.548	2.47 ± 0.16	0.519	2.35 ± 0.16	0.115
	MSG	2.75 ± 0.15		2.75 ± 0.16		2.81 ± 0.15	

Data presented as mean ± standard error (SE)

In blood test, *n* = 51 (Control group) and *n* = 51 (MSG group) at baseline and intervention, and *n* = 49 (Control group) and *n* = 45 (MSG group) at follow-up.

In physical examination, *n* = 62 (Control group) and *n* = 67 (MSG group) at baseline and intervention, and *n* = 62 (Control group) and *n* = 66 (MSG group) at follow-up

In daietary survey, *n* = 51 (Control group) and *n* = 56 (MSG group) at baseline and intervention, and *n* = 47 (Control group) and *n* = 52 (MSG group) at follow-up

In daily performance, *n* = 68 (Control group) and *n* = 69 (MSG group) at baseline and intervention, and *n* = 66 (Control group) and *n* = 69 (MSG group) at follow-up

^a^Probability values are for the comparison between MSG group and control group at the baseline

^b^Probability value are mean change from baseline comparison between MSG group and control group

^c^Significant difference between baseline and post-intervention (*p* < 0.05)

^d^Significant difference between baseline and post-follow-up (p < 0.05)

The frequency of defecation in the MSG group was 0.61 ± 0.05/day and 0.58 ± 0.05/day during the first and last 4 weeks, respectively; the corresponding values in the Control group were 0.53 ± 0.04/day and 0.60 ± 0.05/day, respectively. There were no significant changes observed in the frequency of defecation during the intervention period in either of the groups (*p* = 0.597 and 0.439, respectively). The frequency of diarrhoea in the MSG group was 0.027 ± 0.008/day and 0.021 ± 0.009/day during the first and last 4 weeks, respectively; the corresponding values in the Control group were 0.019 ± 0.007/day and 0.008 ± 0.005/day, respectively. There were no significant changes observed in the frequency of diarrhoea during the intervention period in either of the groups (*p* = 0.372 and 0.212, respectively).

### Cognitive symptoms according to the TDAS and GBSS scores

**Fig. 2:**
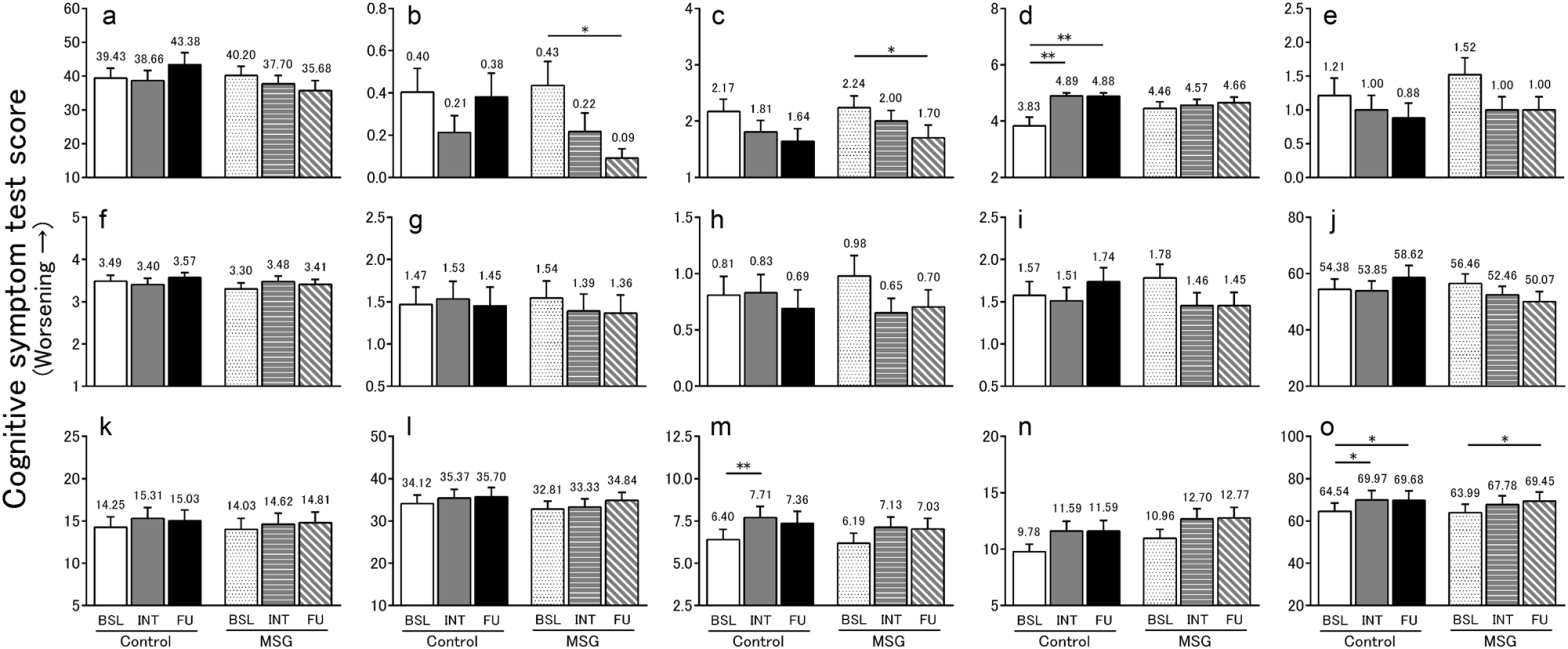
TDAS score (**a**–**j**) and GBSS score (**k**–**o**). The TDAS score indicated (**a**) word recognition, (**b**) following a command, (**c**) visual-spatial perception, (**d**) accuracy of the order of a process, (**e**) naming fingers, (**f**) orientation, (**g**) money calculation, (**h**) object recognition, (**i**) clock time recognition and (**j**) total score. The GBSS score indicated (**k**) motor functions, (**l**) intellectual functions, (**m**) emotional functions, (**n**) symptoms common in dementia syndromes and (**o**) total score. All data indicated mean ± standard error (SE), and the number on the error bar is mean value. In TDAS, *n* = 47 (Control group) and *n* = 46 (MSG group) at baseline (BSL) and intervention (INT), and *n* = 42 (Control group) and *n* = 44 (MSG group) at follow-up (FU). In GBSS, *n* = 68 (Control group) and *n* = 69 (MSG group) at baseline (BSL) and intervention (INT), and *n* = 66 (Control group) and *n* = 69 (MSG group) at follow-up (FU). *p < 0.05, **p < 0.01. TDAS Touch Panel-type Dementia Assessment Scale, GBSS Gottfries–Bråne–Steen Scale, MSG monosodium l-glutamate

**Fig. 3:**
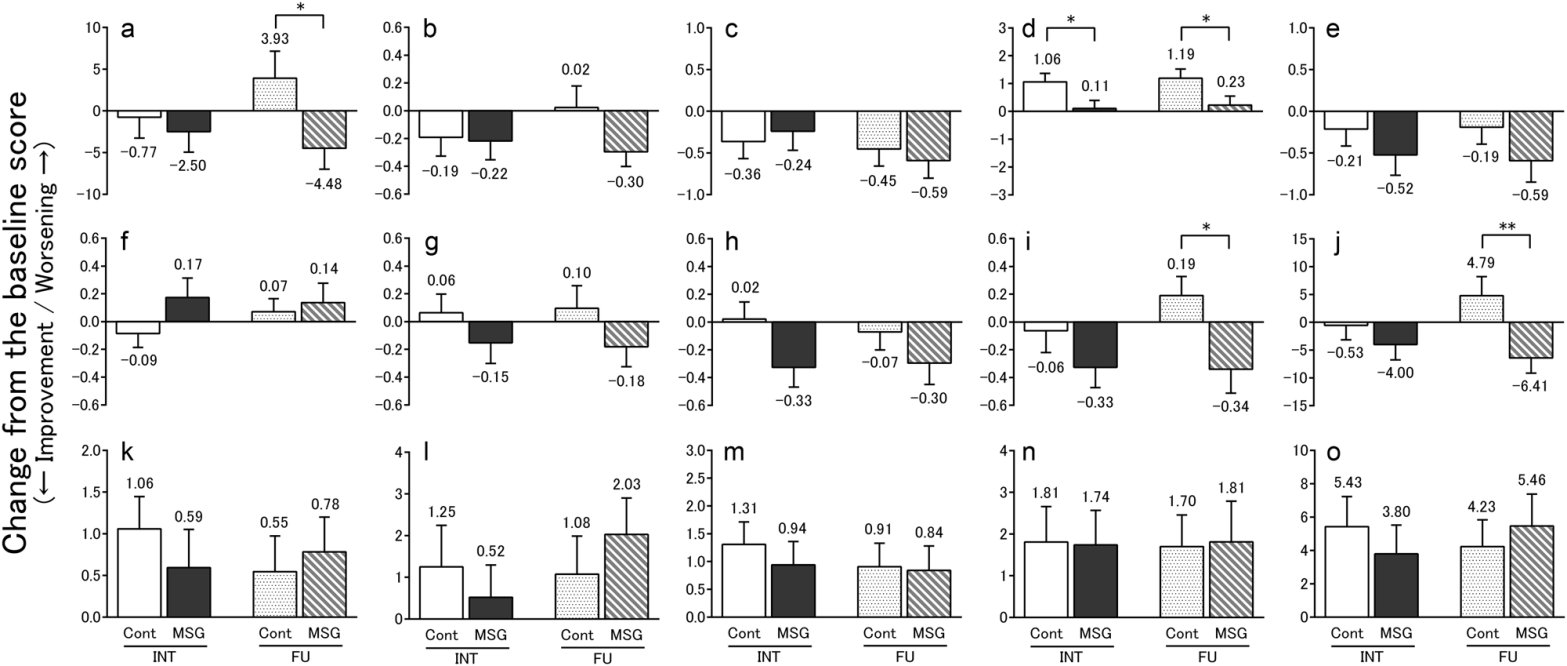
Mean change from baseline in the TDAS score (**a–j**), GBSS score (**k**–**o**). The TDAS score indicated (**a**) word recognition, (**b**) following a command, (**c**) visual-spatial perception, (**d**) accuracy of the order of a process, (**e**) naming fingers, (**f**) orientation, (**g**) money calculation, (**h**) object recognition, (**i**) clock time recognition and (**j**) total score. The GBSS score indicated (**k**) motor functions, (**l**) intellectual functions, (**m**) emotional functions, (**n**) symptoms common in dementia syndromes and (**o**) total score. All data indicated mean ± standard error (SE), and the number on the error bar is mean value. In TDAS, *n* = 47 (Control group (Cont)) and *n* = 46 (MSG group (MSG)) at intervention (INT), and *n* = 42 (Control group (Cont)) and *n* = 44 (MSG group (MSG)) at follow-up (FU). In GBSS, *n* = 68 (Control group (Cont)) and *n* = 69 (MSG group (MSG)) at intervention (INT), and *n* = 66 (Control group (Cont)) and *n* = 69 (MSG group (MSG)) at follow-up (FU). *p < 0.05, **p < 0.01. TDAS Touch Panel-type Dementia Assessment Scale, GBSS Gottfries–Bråne–Steen Scale, MSG monosodium l-glutamate

The results of the TDAS and GBSS scores are shown in Fig. 2a–o, and changes in these scores from baseline are shown in Fig. 3a–o. Changes in the total TDAS scores did not differ significantly between the groups after the intervention. However, after the 4-week follow-up, the total scores improved significantly in the MSG group relative to the Control group (*p* < 0.01; Fig. 3j). In contrast, changes in the total GBSS scores from baseline did not differ significantly between the groups after the intervention and follow-up. Of note, these scores worsened significantly in both the MSG and Control groups after follow-up (*p* < 0.05 and 0.05, respectively; Fig. 2o). Regarding the Control group, the total GBSS score worsened significantly after the intervention compared with that reported prior to the intervention (*p* < 0.05; Fig. 2o).

Regarding the TDAS sub-items, the score of the ‘accuracy of the order of a process’ deteriorated in the Control group (*p* < 0.01; Fig. 2d), and the difference between the groups was statistically significantly (*p* < 0.05; Fig. 3d). Furthermore, in the post-follow-up assessment, the score for ‘word recognition’ and ‘clock time recognition’ improved significantly in the MSG group relative to the Control group (*p* < 0.05 and 0.05, respectively; Fig. 3a, i).

Regarding the GBSS sub-items, changes in scores from baseline did not differ significantly between the groups. However, the emotional functions worsened significantly in the Control group after the intervention compared with that reported prior to the intervention (*p* < 0.01; Fig. 2m).

### Palatability and behaviour questionnaire

The results of the palatability survey and daily performance questionnaire are presented in Table 2. Regarding changes from the baseline of palatability survey, the strength of flavour increased significantly in the MSG group relative to the Control group after the intervention (*p* = 0.022). Regarding daily performance, the maintenance of posture during mealtime improved significantly in the MSG group at the post-follow-up survey, compared to the pre-intervention survey (*p* < 0.05). However, the changes from baseline did not differ significantly between the groups.

### Correlation of the TDAS and palatability survey outcomes

**Fig. 4:**
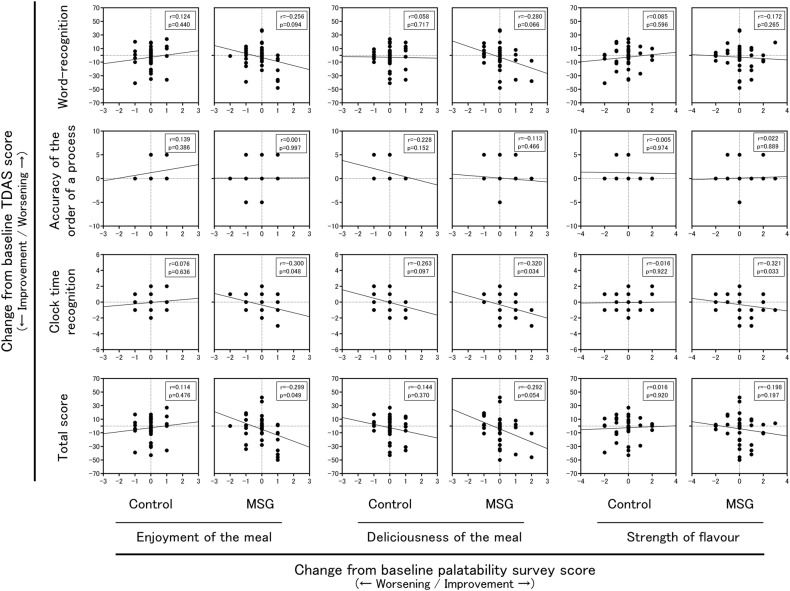
The correlation between TDAS and the results of the palatability survey prior to and after the intervention. *r*, correlation coefficient; TDAS Touch Panel-type Dementia Assessment Scale, MSG monosodium l-glutamate

Figure 4 presents changes in TDAS and scores of the palatability survey from pre-intervention to post-intervention. We selected three TDAS sub-items, namely ‘word recognition’, ‘accuracy of the order of a process’ and ‘clock time recognition’, for which changes from baseline to post-follow-up assessment differed significantly between the groups. In the MSG group, the TDAS total scores correlated significantly with enjoyment of the meal (*r* = −0.299, *p* = 0.049) and tended to correlate with deliciousness of the meal (*r* = −0.292, *p* *=* 0.054). Analysis of the sub-items of TDAS showed that, in the MSG group, there was a significant correlation between ‘clock time recognition’ and enjoyment of the meal (*r* = −0.300, *p* = 0.048), deliciousness of the meal (*r* = −0.320, *p* = 0.034) and strength of flavour (*r* = −0.321, *p* = 0.033) and a tendency towards correlation between ‘word recognition’ and deliciousness of the meal (*r* = −0.280, *p* = 0.066). In contrast, in the Control group, there was no significant correlation observed between the TDAS scores and the results of the palatability survey.

## Discussion

Regarding changes from baseline, the post-follow-up score of total TDAS, ‘word recognition’ and ‘clock time recognition’ improved significantly in the MSG group compared with those observed in the Control group. A previous study on rats reported that the forebrain regions, including the hippocampus, responded to intragastric administration of MSG [26], and stimulation by MSG is thought to convey signals to the brain via the vagus nerve [27]. ‘Word recognition’ is used specifically for the evaluation of hippocampal function. Thus, ingestion of MSG may activate the hippocampus and consequently improve memory. However, we note that in rats, the forebrain regions were also found to respond, albeit less strongly, to intragastric administration of NaCl [26]. In other words, the ability of both MSG and NaCl to stimulate the hippocampus may account for the lack of a significant difference between groups in the change in total TDAS scores from baseline to the post-intervention time point.

In this study, the total GBSS scores deteriorated in both groups after the follow-up period. The GBSS, measuring the degree and determining the profiles of dementia syndromes, was designed for administration by physicians, psychologists, and registered nurses. Although the GBSS has been verified as valid and highly reliable [28], its outcomes are known to depend on the psychosocial intervention of the evaluator [29]. In the present study, this assessment was performed by the major caregivers of the subjects, and the preconceptions and skills of the evaluator were not standardised. It is possible that the preconception of the evaluator that dementia is a progressive disease may affect the GBSS score. In addition, the GBSS evaluation items include cognitive functions and behavioural symptoms. Hence, it is possible that a divergence may have occurred between the results of TDAS and those of GBSS.

A previous study reported that patients with dementia had lower weights as a result of their impaired mental states, which could affect healthy dietary intake and exercise and might also correlate with dysphagia, which could affect dietary intake [30]. Furthermore, a correlation between low BMI and reduced cognitive capacity was identified in patients with dementia [31], and low BMI and weight loss are known risk factors for mortality among the elderly [32]. In this study, the value of change in BMI from baseline increased significantly in the MSG group after the intervention. However, the change in BMI from baseline did not differ significantly between the MSG and Control groups. Ingestion of MSG increases secretion of saliva [15] and gastric juice [16], promoting swallowing and digestion [17].

Although a previous study showed improved daily performance among elderly individuals ingesting MSG [21], the present study including patients with moderate to severe dementia, did not obtain similar results. Patients with dementia are limited with regards to activities of daily living, and these limitations progress with disease severity [33, 34]. The lack of improvement in daily performance may be attributed to this factor. However, the significant correlations detected between changes in total TDAS scores from baseline to post-intervention assessment with enjoyment of the meal suggest that increased interest for meals exerts a beneficial effect on cognitive function.

Although MSG enhances umami, we must also consider that the taste function was enhanced by MSG ingestion. Subjects in this study tended to have low baseline levels of zinc in the serum of 61.8 ± 1.4 µg/dL in the MSG group, 63.5 ± 1.3 µg/dL in the Control group. Although the changes in serum zinc levels from the baseline did not differ significantly between the groups at the post-intervention and post-follow-up time points, the serum zinc levels in the MSG group increased at post-intervention. Intragastric administration of MSG stimulates upper gut motility and accelerates gastric emptying via the vagus nerve [35], and the increased digestive absorption may lead to increase in the absorption of zinc from the intestine. Zinc plays an important role in taste bud homoeostasis [36], and patients with taste disorders have exhibited significant improvements in gustatory sensitivity after treatment with a zinc-containing compound [37]. Therefore, elevated levels of zinc in the serum may have led to regeneration of taste buds. Moreover, MSG acts on the umami receptors, T1R1/T1R3, expressed on taste buds [38]. Thus, subjects became more aware of the flavours of foods and experienced enhanced transmission of taste signals to the brain.

A few limitations of this study must be considered. Firstly, assessment may not have been completed in all subjects. For example, the TDAS, which is self-administered test using a computer, could not be completed by subjects with severe dementia. Secondly, improvements in cognitive function were observed after 4 weeks without MSG ingestion. Initially, we believed that cognitive function would deteriorate when MSG was removed, and designed this study to support our hypothesis. In rats, the average lifespan of a taste bud cell is 250 ± 50 h [39]. It was suggested that zinc deficiency may delay the replacement of taste bud cells [40]. Although we cannot make a clear conclusion, taste bud regeneration in response to increased zinc absorption during the MSG intake period might have appeared as a sustained effect even after MSG discontinuation. In addition, a previous study involving patients with AD found that scores from the ADAS, which provides a basis for the TDAS, increased by 9–11 points over a 1-year period [41]. Therefore, because we did not observe large decreases in cognitive function during the 12-week period, even in the Control group, this may account for the lack of significant difference observed between the groups in terms of changes in total TDAS scores from baseline to post-intervention assessment. Furthermore, we evaluated the intake of meals containing MSG or NaCl, but were unable to evaluate the overall intake of the provided food. Subjects were recruited from multiple facilities, thus, we were unable to accurately standardise the amount and content of meals.

In conclusion, we examined the effect of continuous ingestion of MSG on cognitive symptoms and observed no significant improvements in cognitive function from before to after a 12-week dietary intervention. However, subjects in the MSG group exhibited improved cognitive function at a follow-up 4 weeks after discontinuation of MSG ingestion. Subjects who reported that interest for meals was improved by MSG also experienced greater improvements in cognitive function. Meals are consumed daily and individuals with a satisfactory diet are reported to have better cognitive function [42]. Therefore, it is important not only ingestion of MSG but also a balanced diet. In the future, we consider it necessary to examine the effects of more longer-term ingestion of MSG on cognitive function.
